# Perform hand hygiene and the doors will open – the effectiveness of new system implementation on paediatric intensive care unit visitors’ handwashing compliance

**DOI:** 10.1017/S0950268821002582

**Published:** 2021-12-17

**Authors:** Eli Shapiro, Keren Mahlab-Guri, Eric Scheier, Pnina Ciobotaro, Alex Guri

**Affiliations:** 1 Kaplan Medical Centre, Rehovot, Israel; 2 The School of Medicine, The Hebrew University and Hadassah Medical Centre, Jerusalem, Israel

**Keywords:** Hand hygiene, infectious disease control, intensive care units, visitors to patients

## Abstract

Hand hygiene (HH) performance on entering intensive care units (ICUs) is commonly accepted but often inadequately performed. We developed a simple, inexpensive module that connects touchless dispensers of alcohol sanitiser (TDAS) to the automatic doors of a paediatric ICU, and assessed the impact of this intervention on HH compliance of hospital staff and visitors. A prospective observational study was conducted over a 3-week period prior to the intervention, followed by a 4-week period post intervention. HH performance was monitored by a research assistant whose office location enabled direct and video-assisted observation of the ICU entrance. A total of 609 entries to the ICU was recorded. Overall HH performance was 46.9% (92/196) before and 98.5% (406/413) after the intervention. Our findings suggest that HH performance on entering an ICU can be improved via a mechanism that makes operation of an automatic door dependent on use of a TDAS system, and thus contribute to infection control.

Hand hygiene (HH) is a universally recognised critical infection-control measure as efficient hand antisepsis reduces the incidence of outbreaks of health-care-associated infections (HCAIs), which can often be traced to poor practice of health-care workers (HCWs) [1]. Visitors may also be vectors for pathogen transmission in the ward and potentially import community-associated antimicrobial-resistant organisms [2]. In the paediatric setting, interventions to improve HH compliance among parents have been shown to reduce the incidence of viral HCAI in a neonatal intensive care unit (ICU) [3]. Although visitor restriction policies and practices vary in different paediatric facilities [4], the COVID-19 pandemic has raised general awareness of the importance of infection control measures. Increasing hospital staff engagement and HH compliance are therefore key measures for more effective infection control [5].

Technological solutions have been developed to enhance monitoring of HH compliance, but should be accompanied with educational tools [6]. Similarly, accessibility of hand-washing devices does not alone lead to perfect compliance [7], and educational campaigns and signage to inform visitors to hospitals of HH have also proved insufficient [8].

Our innovation connected the physical barrier to entering the paediatric ICU (PICU) (automatic electric doors) with readily accessible touchless dispensers of alcohol sanitiser (TDAS) to assess its impact on HH compliance of PICU visitors and staff before and after implementation of the intervention. A secondary aim was to evaluate the baseline compliance with local HH guidelines in different staff sectors.

The study was performed at the PICU of Kaplan Medical Centre (KMC), affiliated with the Hebrew University of Jerusalem, Israel. The six-bed PICU has four semi-open single rooms and two rooms for patients who need airborne isolation. The main entrance to the unit is through door A (Fig. 1) which can be opened automatically by staff key-card or by the guests' intercom system. Visitors first enter the lobby and then pass into the main area through door B. Alternatively, the visitor can enter the corridor where the staff offices and the parents’ resting rooms are located, and progress to the main area through door C. TDAS systems (Steripower©, Starnberg, Germany) were connected to the opening mechanism of doors B and C via an electronic module developed in-house which initiates automatic door opening after HH has been performed (Supplementary Fig. S1).

**Fig. 1.**
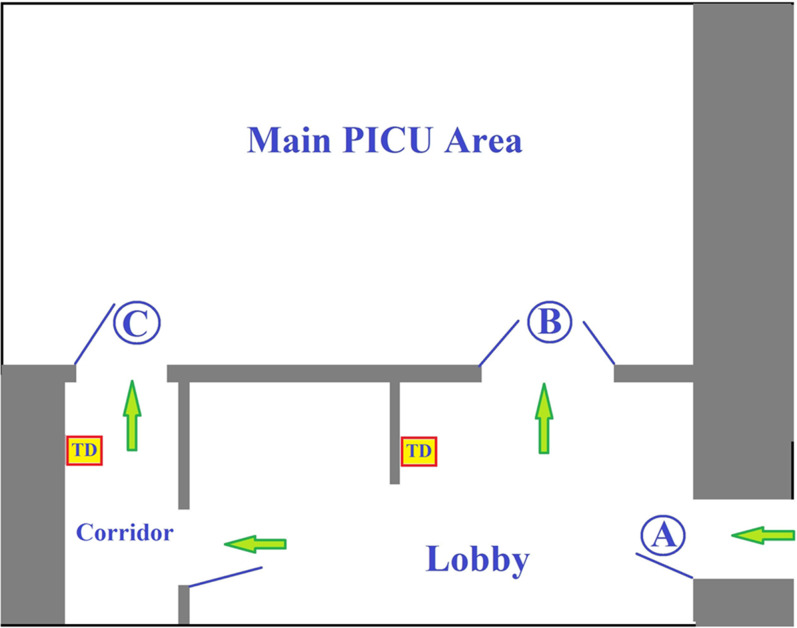
Paediatric intensive care unit schematic plan. PICU, paediatric intensive care unit; TD, touchless dispenser (of alcohol sanitiser).

In order to evaluate the impact of the devices, the connection was not activated in the first stage of the study. Large signs located above the entrance doors instructed visitors to perform HH from dispensers adjacent to the automatic doors before entering the PICU. The PICU secretary, whose office is located in the unit lobby, served as a research assistant for the purpose of the study and monitored HH compliance by staff and visitors using hidden video cameras in the first study stage for 3 weeks. Only the research initiators and members of the institutional review board (IRB) were aware of the study, to prevent the ‘Hawthorne effect’ whereby people improve behaviour only because they are being observed. We included in the study all visitors to the PICU during morning shifts in the study period, and excluded the study authors. Each entry was recorded. In the second stage of the study the connections between the dispensers and the door mechanisms were activated, and HH compliance was monitored for an additional 4 weeks. Hospital staff retained the option to open the doors with a key-card, without activating the TDAS.

The primary outcome was defined as the proportion of individuals who performed HH before entering the PICU. ‘HH performance’ was completed if both of the entering individuals' hands were inserted into the TDAS device. The primary objective was to compare rates of HH compliance in different sectors during the first and second study stages for PICU staff, other hospital HCWs patients' parents and other visitors. Data were analysed for statistical significance by using the Fisher exact test.

During the 7-week study period, a total 609 entries to the PICU were recorded; 196 were logged in the first 3-week stage prior to the intervention, and 413 post intervention (Table 1). Overall HH performance was 46.9% (92/196) before, and 98.5% (406/413), after the intervention. PICU staff performed HH on 81.8% (27/33) of entries before the intervention and all (78) were compliant in the second stage. Notably, the nutritionists proved to be the most compliant with HH practice (100% (8/8)), followed by visiting physicians (65% (22/34)). By contrast, several other staff sectors such as X-ray technicians, ancillary and security staff failed to perform adequate HH before the intervention but only six entries of PICU visitors without HH performance were recorded after the intervention (Table 1).

**Table 1.** HH performance rate on entering PICU before and after the intervention

	Before intervention					After intervention					*P*-value
	HH (*N*)	%	No HH (*N*)	%	Total before	HH (*N*)	%	No HH (*N*)	%	Total after	
PICU staff	27	81.8	6	18.2	33	78	100.0	0	0.0	78	0.0005
PICU visitors (HCWs)											
Physicians	22	64.7	12	35.3	34	75	98.7	1	1.3	76	<0.0001
Nurses	5	23.8	16	76.2	21	41	100.0	0	0.0	41	<0.0001
X-ray technicians	0	0.0	4	100.0	4	4	100.0	0	0.0	4	0.0286
Housekeeping workers	3	25.0	9	75.0	12	6	100.0	0	0.0	6	0.009
Kitchen workers	3	25.0	9	75.0	12	14	93.3	1	6.7	15	0.0008
Nutritionists	8	100.0	0	0.0	8	15	100.0	0	0.0	15	1
Porters	0	0.0	5	100.0	5	23	92.0	2	8.0	25	0.0001
Maintenance workers	0	0.0	10	100.0	10	16	100.0	0	0.0	16	<0.0001
Educational staff	7	46.7	8	53.3	15	44	100.0	0	0.0	44	<0.0001
Pharmacists	1	14.3	6	85.7	7	10	100.0	0	0.0	10	0.0006
Security staff	0	0.0	1	100.0	1	3	75.0	1	25.0	4	0.4
Ventilation technicians	0	0.0	2	100.0	2	2	66.7	1	33.3	3	0.4
Other visitors	4	32.7	5	55.6	9	10	100.0	0	0.0	10	0.0108
Total HCWs (visitors)	53	37.9	87	62.1	140	263	97.8	6	2.2	269	<0.0001
Patients' parents	12	52.2	11	47.8	23	66	100.0	0	0.0	66	<0.0001
Total (visitors and staff)	92	46.9	104	53.1	196	407	98.5	6	1.5	413	<0.0001

HH, hand hygiene; PICU, paediatric intensive care unit; HCW, health-care workers.

Previous studies have reported variable HH compliance rates among ICU staff. Nishimura *et al*. used video camera monitoring of HH compliance rates of all visitors entering an ICU over a 7-day period and reported rates of 71% for ICU staff, 74% for other staff and 95% for patients’ visitors [9]. By comparison, only 34% of hospital personnel in two US hospitals were reported to comply with HH performance according to the WHO's standardised method [10].

Our hospital policy requires all HCWs and visitors to perform HH before entering the ICU. Surprisingly, only half of the patients' parents (12/23) did so prior to the study intervention. Indeed, compliance rates as low as 10% by hospital visitors, despite proper signage and easy access to alcohol dispensers, has been reported. Similarly, as recorded here and by others [10], compliance of other HCWs was less than that of patients' parents (37.9% of entries) [10].

The prevalence of HCAI remains high even in developed countries with stringent infection-control measures. Most nosocomial pathogens are transmitted via the hands of health-care personnel and caregivers, from contaminated surfaces or equipment in the hospital environment [11]. The introduction of HH institutional policies has been proven to reduce HAIs and to be cost-effective [12], and the placing of barriers with mandatory HH performance should be a component of this policy.

Barriers to adherence with HH recommendations include structural aspects, knowledge gaps and especially a lack of time in daily routine practice. The latter issue is pivotal [9] as HH performance using TDAS can be measured in seconds. Similarly, siting a mandatory TDAS usage barrier at the entrance to the PICU should not directly affect compliance with the ‘five moments for hands hygiene’ model but is complementary to the process. This intervention reminds visitors that they are entering a ‘clean zone’ and diminishes baseline hand contamination with minimal time investment.

Our study has some limitations. First, the pre-post study design can be affected by unexpected biases. We made our best effort to keep the study secret. Second, the connection of the door entry mechanism to the TDAS may allow groups of people to enter at the same time without all performing HH. Third, the study wasn't planned to assess the impact of the intervention on the incidence of HAIs, and the load of potentially pathogenic bacteria on the hands of visitors was not evaluated. Nevertheless, this study shows how readily HH compliance can be achieved. The groups (before and after the intervention) differed in size, as we wished to ensure that the effect was sustainable, and data collection was therefore continued for a longer-than-planned period of time. Occasional changes in volume of PICU activity also increased the number of observations between pre- and post-intervention periods. Moreover, data collection was performed only during the day-shifts and not on weekends, because of the logistical issues.

In conclusion, our study shows that significant improvement of HH performance on entering the PICU by hospital staff and other visitors was achieved subsequent to implementation of the intervention. The improvement was surprisingly high in hospital staff, since personnel could use key-cards to open the doors. Our findings suggest that HH performance on entering any healthcare facility can be dramatically improved by installing this inexpensive and easy-to use module, and could be extended to out of hospital platforms as part of the global effort to prevent the spread of infectious diseases.

## Data Availability

The data used to support the findings of this study are available from the authors upon request.

## Supplementary material

For supplementary material accompanying this paper visit https://doi.org/10.1017/S0950268821002582. click here to view supplementary material
