# Supplementary figures and images for: Perform hand hygiene and the doors will open – the effectiveness of new system implementation on paediatric intensive care unit visitors’ handwashing compliance
Source: Epidemiol Infect. 2021 Dec 17;150:e3. doi: 10.1017/S0950268821002582 (PMC8755529; doi:10.1017/S0950268821002582)

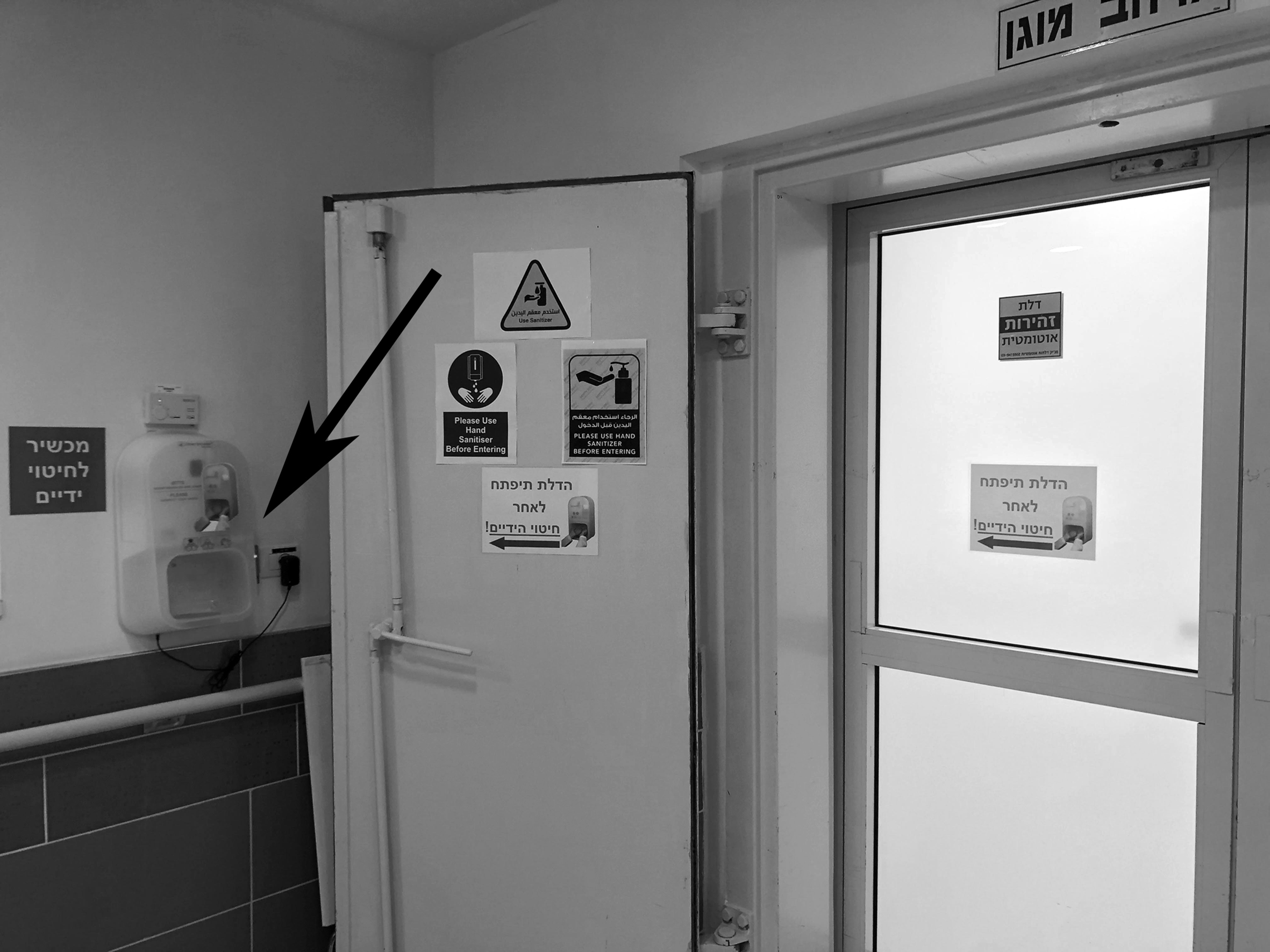

Supplement: Supplementary file 1 [file S0950268821002582sup001.jpg]
